# Capacity building in pediatric surgical neuro-oncology: Assessment of clinical collaboration within limited resources in a low-middle-income country

**DOI:** 10.12669/pjms.41.13(PINS-NNOS).13438

**Published:** 2025-12

**Authors:** Ahtesham Khizar, Rahat-Ul-Ain, Haseeb Mehmood Qadri, Arooj Kiran, Zia Ul Rehman Najeeb, Hassaan Zahid

**Affiliations:** 1 Ahtesham Khizar, MBBS, FCPS (Neurosurgery). Punjab Institute of Neurosciences, Lahore, Pakistan; 2 Rahat-Ul-Ain, MBBS, FCPS (Pediatric Medicine), FCPS (Pediatric Hematology/Oncology),Fellowship in Pediatric Neuro-oncology, The Children’s Hospital and Institute of Child Health, Lahore, Pakistan; 3 Haseeb Mehmood Qadri, MBBS. Punjab Institute of Neurosciences, Lahore, Pakistan; 4 Arooj Kiran, MBBS. Punjab Institute of Neurosciences, Lahore, Pakistan; 5 Zia Ul Rehman Najeeb, MBBS. Punjab Institute of Neurosciences, Lahore, Pakistan; 6 Hassaan Zahid, MBBS, FCPS (Neurosurgery), MS (Pediatric Neurosurgery). Punjab Institute of Neurosciences, Lahore, Pakistan

**Keywords:** Capacity building, CNS neoplasms, Developing country, Pediatrics, Surgical oncology

## Abstract

**Objective:**

To analyze the impact of a visiting consultant pediatric neuro-oncologist on the outcome of pediatric patients with brain tumors in a health care system with no consultant pediatric neuro-oncologist.

**Methodology:**

This retrospective study was conducted at the Punjab Institute of Neurosciences, Lahore, Pakistan, involving pediatric patients with brain tumors from January 2023 to January 2025. Patients were assessed by a visiting pediatric neuro-oncologist. These patients were followed for one year, and outcomes were observed.

**Results:**

This study included 45 patients who were assessed by a visiting neuro-oncologist; 82.22% (37) underwent craniotomy and excision, and 8.89% (4) had a biopsy. In histopathology, the most common tumor was glioma, with 37.78% (17) of patients, and 24.44% (11) were referred for adjuvant therapy by the visiting neuro-oncologist. After a one-year follow-up, 13 patients were lost to follow-up (LTFU) during their treatment, with an overall LTFU rate of 28.89%. Of those with complete follow-up, 90.63% of patients were healthy without any new neurological deficits.

**Conclusion:**

In countries with limited resources and few specialists, particularly pediatric neuro-oncologists, such a collaboration of shared care can impact the management and outcome of the patients without pouring more resources. However, LTFU remains a major challenge to assess complete disease courses and management outcomes in clinical practice.

***List of Abbreviations:*** **AMC:** Academic medical center, **CNS:** Central nervous system, **CSF:** Cerebrospinal fluid, **DIPGs:** Diffuse intrinsic pontine gliomas, **HICs:** High-income countries, **HGGs:** High-grade gliomas, **HSS:** Health strengthening systems, **IRB:** Institutional Review Board, **LGGs:** Low-grade gliomas, **LMICs:** Low-middle-income countries, **LTFU:** Lost to follow-up, **PACS:** Picture archiving and communication system, **PBTES:** Pakistan Brain Tumor Epidemiology Study, **PNSO:** Pediatric neurosurgical oncology, **QoL:** Quality of life, **SDGs:** Sustainable Development Goals, **SOL:** Space-occupying lesion.

## INTRODUCTION

Pediatric neurosurgical oncology (PNSO), a field focused on the management of central nervous system (CNS) cancers in individuals aged 0-18 years, holds immense importance globally. Pediatric brain tumors represent the leading cause of cancer-related mortality in high-income countries (HICs).^1^ However, the situation is even more challenging in lower-middle-income countries (LMICs) such as Pakistan, where resources and specialized facilities for PNSO care are scarce.^2^ The limited availability of multidisciplinary expertise, specialized infrastructure, and diagnostic and therapeutic tools in LMICs exacerbates the burden of pediatric CNS tumors.

Globally, the neurosurgical community, alongside institutions and individuals, has taken significant strides toward addressing disparities in access to neurosurgical care. Efforts to increase capacity in LMICs often involve partnerships with HICs that sponsor capacity-building activities such as workshops, teaching operations, didactic sessions, and collaborative research. These initiatives aim to strengthen healthcare systems and promote equitable access to PNSO care.^2^

Pediatric CNS tumors are the most common solid neoplasms in children, representing approximately 20% of all childhood cancers and ranking second in frequency after leukemia. They are a leading cause of cancer-related mortality among children. Tumor subtypes vary widely, with low-grade gliomas (LGGs) accounting for approximately one-third of pediatric brain tumors. High-grade gliomas (HGGs) are less common, comprising about 10% of pediatric CNS tumors, yet they carry a significantly poorer prognosis. Other common pediatric CNS tumor types include ependymomas, embryonal tumors (e.g., medulloblastoma), and diffuse intrinsic pontine gliomas (DIPGs).^3^

Despite advances in surgical techniques and adjuvant therapies, the prognosis for malignant pediatric CNS tumors remains suboptimal, particularly in LMICs. Survival rates in economically developed regions, such as the United States and Europe, range between 56% and 63%, while LMICs like South Africa and India report significantly lower survival rates of 46.4% and 27%, respectively.^4^

Capacity building in PNSO is not solely a matter of increasing workforce numbers. It also requires improvements in quality, organization, and resource management. Strategies such as enhancing workforce efficiency and providing targeted training can help develop a skilled workforce capable of delivering comprehensive, patient-centered cancer care in resource-constrained settings.^5^

Our pediatric neurosurgery section comprises one assistant professor of pediatric neurosurgery and one senior registrar at the moment. We provide services for all pediatric neurosurgery cases. To improve outcomes and capacity in a limited-resource public sector hospital, we employed the services of a pediatric neuro-oncology specialist, underscoring the need for context-specific solutions in PNSO care. Additionally, studies like those by Gringmuth et al. highlight the potential of multimodal therapies to enhance survival rates, further emphasizing the importance of research-driven capacity building.^6^

## METHODOLOGY

A retrospective, descriptive, and qualitative study was designed to evaluate the impact of a neuro-oncology consultant in a neurosurgical setup without an in-house oncology department. By reviewing past patient records and conducting qualitative assessments, the study aimed to document the consultant’s collaborative role in building capacity for neurosurgical oncology within limited resources. The study was conducted at the Department of Neurosurgery-Pediatric Neurosurgery Section, Punjab Institute of Neurosciences, Lahore, Pakistan, in collaboration with a pediatric neuro-oncology consultant from a neighboring pediatric specialty hospital, The Children’s Hospital and Institute of Child Health, Lahore, Pakistan. The data was collected between January 2023 and January 2025. The study population consisted of pediatric patients with CNS tumors.

### Ethical Approval:

Patient confidentiality was maintained by anonymizing all data during analysis and reporting. Institutional Review Board (IRB) approval was obtained for this retrospective study (IRB Ref # 2072/IRB/PINS/Approval/2025, dated February 12, 2025).

### Inclusion criteria:

- All pediatric patients (<18 years) with suspected or diagnosed CNS tumors.
- Patients whose records were complete with relevant clinical, surgical, and follow-up details.

### Exclusion criteria:

- Adult patients (>18 years).
- Patients with incomplete records or missing follow-up data.

With non-probability, purposive sampling, the data were extracted retrospectively from patient records, including clinical notes, operative reports, follow-up documentation, and hospital transfer records. Additional information was obtained from the institutional picture archiving and communication system (PACS) for radiological assessments and the histopathology laboratory. Demographics like age, gender, and socioeconomic background; clinical information on diagnosis, tumor type, and location; surgical interventions; the role of the pediatric neuro-oncology consultant, including pre-operative assessment, intraoperative considerations, post-operative adjuvant therapy, and follow-up plans; patient outcomes; collaboration dynamics; and qualitative assessment of the impact of collaboration were noted. The quantitative data was analyzed using descriptive statistics. The qualitative component included a thematic analysis of the consultant’s role, extracted from clinical notes and team discussions.

## RESULTS

The results of our study showed the mean age with standard deviation (SD) of 10.56±4.56 years and the male percentage of 53.33% (24), while the female percentage was 46.67% (21) (Table-I).

**Table-I:** Descriptive analysis of demographic and location presentation of pediatric patients with intracranial space-occupying lesions (SOLs).

Variables	Values
Mean age ± SD (y)	10.56 ± 4.56
Gender distribution, M/F, % (n)	24 (53.33%) / 21 (46.67%)
Site of SOL, % (n)	Posterior fossa - 13 (28.89%)
	Sellar/suprasellar - 12 (26.67%)
	Thalamic - 4 (8.89%)
	Occipital lobe - 3 (6.67%)
	Frontal lobe - 2 (4.44%)
	Intraventricular - 2 (4.44%)
	Parietal lobe - 2 (4.44%)
	Temporal lobe - 2 (4.44%)
	Temporo-parietal lobes - 2 (4.44%)
	Fourth ventricle - 1 (2.22%)
	Orbital - 1 (2.22%)
	Parieto-occipital - 1 (2.22%)

Overall, the most common group of tumors was gliomas, 17 cases (37.78%) out of the total 45 pediatric patients with intracranial SOLs. Craniotomy and excision (C & E) were done in 37 cases (82.22%), biopsy in four cases (8.89%), and cerebrospinal fluid (CSF) diversion procedures in four cases (8.89%). CSF diversion procedures were required in three patients with craniopharyngioma; Ommaya reservoir in two cases. Overall, 11 patients (24.44%) were referred by the pediatric neuro-oncologist for adjuvant therapy in the form of chemotherapy, radiotherapy, or a combination of both. Thirteen patients (28.89%) were lost to follow-up during the due course of treatment; however, seven (15.56%) never followed back immediately postoperatively. Of those who had regular follow-ups by the end of the first year, 29 were living healthy lives with no new neurological deficits (90.63%), and three had died within one year (9.37%). Details of all cases based on histopathology are given in Table-II. Thirteen patients were lost to follow-up during their treatment, so the overall percentage of LTFU patients was 28.89% of the total 45 patients recruited.

**Table-II:** Descriptive analysis of surgical management, decisions by the pediatric neuro-oncologist, and outcomes at the end of the first year of follow-up with respect to histopathology diagnosis of pediatric patients with intracranial SOLs.

Histopathology Diagnosis	Type of Surgical Management	Decision by Pediatric Neuro-oncologist	Outcome at 1 year
Pilocytic astrocytoma 9 (20%)	C & E: 8	FUHP: 6	HNND: 6
	C & B: 1	FUMRI: 2	LTFU: 3
		ACT: 1	
Low-grade glioma 4 (8.89%)	C & E: 3	FUHP: 1	HNND: 4
	EB: 1	ACT: 2	
		ACRT: 1	
Diffuse midline glioma 1 (2.22%)	C & B	ACRT	Death
Glioblastoma 1 (2.22%)	C & E	FUHP	LTFU
Pilomyxoid astrocytoma 1 (2.22%)	C & B	ACT	HNND
Pilomyxoid xanthoastrocytoma 1 (2.22%)	C & E	ACT	HNND
Craniopharyngioma 9 (20%)	C & E: 6 CSF diversion: 3	FUHP: 2 FUMRI: 7	HNND: 7 Death: 2
Medulloblastoma 5 (11.11%)	C & E: 5	FUHP: 3 ACRT: 2	HNND: 5
Ependymoma 4 (8.89%)	C & E: 3 CSF diversion: 1	FUHP: 1 FUMRI: 1 ART: 2	HNND: 4
Choroid plexus papilloma 2 (4.44%)	C & E: 2	FUHP: 1 FUMRI: 1	HNND: 1 LTFU: 1
DNET 1 (2.22%)	C & E	FUHP	LTFU
LTFU after surgery 7 (15.56%)	C & E: 7	-	-

C & E: craniotomy and excision; C & B: craniotomy and biopsy; CSF diversion: inclusive of ventriculoperitoneal shunt, Ommaya reservoir, and ventriculo-atrial shunt; FUHP: follow-up after histopathology; FUMRI: follow-up with serial MRI brain; ACT: adjuvant chemotherapy; ART: adjuvant radiotherapy; ACRT: adjuvant chemoradiotherapy; HNND: healthy with no neurological deficits; LTFU: lost to follow-up.

## DISCUSSION

Pediatric brain tumors account for 20-25% of pediatric tumors and are the second most common cause of lifelong disability and mortality.^7^ There are various management options, including safe surgical excision, biopsy, and adjuvant therapy. Therefore, managing pediatric intracranial tumors requires an integrated multidisciplinary approach to improve outcomes.^8^ A current issue in global health is the shortage of neurosurgeons and neuro-oncologists to overcome the current burden of neurological diseases. In addition, every year, approximately 22.6 million individuals require neurosurgical consultation, and 13.8 million require surgery.^9^ Neurosurgical diseases make up an important part of the global burden of diseases. Neurosurgical care has been linked to 14 of the 17 Sustainable Development Goals (SDGs), thus highlighting the huge impact that capacity building in neurosurgery can have upon health strengthening systems (HSS) initiatives.^9^ Hence, neurosurgical and oncological capacity building is required to overcome the shortage in lower- and middle-income countries.

Our study found that 90.63% (29) of patients had a healthy life with no new neurological deficits, 9.37% (3) died within the first year of follow-up, and 28.9% (13) were lost to follow-up. Our findings were consistent with a retrospective longitudinal study conducted by Ndubuisi et al. in 2018, in which 55.6% of patients survived. The most prevalent malignancies in this study were glioma 39.3% (11), medulloblastoma 50% (13), and craniopharyngioma 39.3% (11).^7^ These findings are very identical to ours, which found pilocytic astrocytoma 20%(9), craniopharyngioma 20% (9), and medulloblastoma 11.11% (5) as the most prevalent tumors. However, the better survival in our study may be because of the small sample size, the best follow-up care provided by our neurosurgery team along with an oncologist, and the patient being kept in close contact and being given a personalized care plan on follow-up visits. The preoperative functional status of patients and the extent of tumor resection also proved to be the main factors for better outcomes because patients who underwent complete excision showed better survival rates at one year. Gliomas represented the most frequent tumor type among patients who received craniotomy and excision as treatment, but many patients disappeared from follow-up. The results demonstrate that early detection combined with full surgical removal of tumors and organized post-surgical care leads to better long-term results.^7^ Furthermore, the type of tumor also affected the mortality of the patients. Glioblastoma followed by anaplastic glioma has the highest early mortality in the tumor subtypes.^10^

In a healthcare setting with no specialized care for pediatric neuro-oncology, a visiting consultant can play an important role by providing advanced management options and being the patient’s life changer. The involvement of a visiting pediatric neuro-oncologist in our center had a significant impact on patients’ lives. Postoperatively, all patients (45), along with histopathology, were reviewed by the pediatric neuro-oncologist, and 24.44% (11) of patients were referred for adjuvant therapy in the form of chemotherapy, radiotherapy, or a combination of both. A retrospective Nigerian study done by Usman et al. in 2016 showed a significant impact of a visiting consultant neurosurgeon who reviewed 1204 patients; 18.8% (226) underwent surgical intervention, with a mortality rate of 13.5% (18), which indicated improvement from other rural settings where no visiting consultant was available.^11^ Another study showed that the integration of oncological specialists improved patient care management, patient satisfaction, outcomes, and institutional health with minimum cost.^12^

The problem of loss to follow-up (LTFU) is a significant issue that affects the quality of care and the validity of research in many clinical areas. A loss to follow-up rate of 18% was reported in a study done in a large academic medical center (AMC), and the study showed that age, physician transitions, and social vulnerability were the main factors that contributed to this. This is intriguing since it demonstrated that patients with more complicated medical and psychological disorders were less likely to be lost to follow-up, possibly because they require more health care and have stronger connections with their clinicians. The findings indicate that the systems in AMCs are faulty. The doctor-patient relationship is short-term, and there are difficulties in accessing care, and thus there is a need to develop team-based care and flexible methods.^13^ In general, the literature shows that large LTFU rates, above 20%, can invalidate the results of the studies, introduce selection bias, and obscure treatment results. Nevertheless, LTFU is frequently underreported, and no standardized guidelines exist for dealing with it or assessing its impact.^14^ In the field of neurosurgical oncology in low- and middle-income countries such as Pakistan, the problem is worse. Results of the Pakistan Brain Tumor Epidemiology Study (PBTES) showed that 40% of patients were lost to follow-up, and most of these patients were unable to receive adjuvant therapies such as chemotherapy (76.5%) and radiotherapy (84.1%). Notably, patients from lower socioeconomic statuses, married adults aged 19-49, and those treated at the public sector hospitals were overrepresented. This is different from the pattern that is described in high-income countries, where the elderly, unmarried, and rural patients are the most vulnerable. The differences may be attributed to the health systems, financial systems, and cultural systems of the country. In general, these findings stress the need for specific interventions, such as centralized health registries, improving care continuity during physician transitions, and equal access to care, especially in vulnerable groups.^15^ At the one-year follow-up, 28.89% (11) of enrolled patients were lost to follow-up. Although this rate is relatively low compared to PBTES, contributing factors likely included illiteracy, particularly low health literacy, cultural beliefs, financial limitations, transportation challenges, and the high cost of treatment.

### Limitations:

This study has several limitations that must be acknowledged. Firstly, this was a retrospective study with a small sample size. This may limit the generalizability of the findings to broader pediatric neuro-oncology populations, and the retrospective nature of the study is subject to documentation bias and lacks control over confounding variables that could influence outcomes. Secondly, our study assessed only one-year survival and neurological status, without evaluating long-term quality of life (QoL) or functional outcomes.

## CONCLUSION

This study highlights the positive impact of involving a visiting pediatric neuro-oncologist in a resource-limited neurosurgical setting. The integration of a specialist team-based approach improved surgical decision-making, postoperative care, and referrals for adjuvant therapy, resulting in favorable short-term outcomes. However, a significant LTFU remains a major barrier to long-term care and outcome assessment. Strengthening multidisciplinary collaboration, improving patient tracking, and addressing socioeconomic barriers are essential. These findings support the need for scalable, collaborative models to enhance pediatric neuro-oncology care in LMICs, without necessitating substantial resource expansion.

### Recommendations:

The integration of a visiting pediatric neuro-oncologist demonstrated a measurable impact on patient outcomes. To ensure continuity and comprehensive care, dedicated pediatric neuro-oncology units should be established within tertiary care centers. These units should include trained neurosurgeons, pediatric oncologists, radiologists, pathologists, and supportive care specialists to manage CNS tumors collaboratively. With an LTFU rate of 28.89%, there is a critical need for a centralized pediatric neuro-oncology registry. Such a system would facilitate consistent patient tracking, reduce follow-up attrition, and improve the evaluation of long-term outcomes. The registry should be accessible across institutions and integrated with electronic health records to enhance coordination of care. Addressing barriers such as low health literacy, cultural misconceptions, and financial constraints requires targeted community-based interventions. Educational campaigns, caregiver counseling, and the inclusion of community health workers can improve treatment adherence and reduce LTFU.

Given the demonstrated benefit of specialist input, telemedicine should be adopted to provide regular access to pediatric neuro-oncology expertise, especially in hospitals without in-house consultants. Virtual tumor boards and remote consultations can support local clinical decision-making and ensure patients receive guideline-based care irrespective of geographical limitations. While this study provides insight into short-term survival and neurological status, future research should focus on long-term functional outcomes and QoL. Prospective studies incorporating validated QoL instruments (e.g., PedsQL) will help to assess the broader impact of interventions and guide improvements in survivorship care.

### Author`s Contribution:

**AK:** Conception and design of study, critically reviewed the manuscript.

**RUA, HMQ:** Data analysis and data interpretation drafted and critically reviewed the manuscript.

**AK and ZURN:** Data acquisition and data interpretation, drafted the manuscript.

**HZ:** Supervision, conception and design of study, critically reviewed the manuscript.

All the authors have read and approved the final manuscript for the publication.

All agreed to be accountable for all aspects of the work related to accuracy or integrity.
